# Plant hormones in interactions with the environment

**DOI:** 10.1007/s11103-016-0501-8

**Published:** 2016-06-29

**Authors:** Eva Benková

**Affiliations:** Institute of Science and Technology Austria, Klosterneuburg, Austria

Plants are continuously exposed to a myriad of external signals such as fluctuating nutrients availability, drought, heat, cold, high salinity, or pathogen/pest attacks that can severely affect their development, growth, and fertility. As sessile organisms, plants must therefore be able to sense and rapidly react to these external inputs, activate efficient responses, and adjust development to changing conditions. In recent years, significant progress has been made towards understanding the molecular mechanisms underlying the intricate and complex communication between plants and the environment. It is now becoming increasingly evident that hormones have an important regulatory role in plant adaptation and defense mechanisms.

This special issue is dedicated to plant hormones in interactions with the environment. It brings together a selection of articles that discuss the most recent advances in how plants sense and integrate the external inputs into optimal physiological and developmental responses, and in particular, the role of plant hormones as internal mediators of the interaction between plants and their surrounding environment. The topical reviews focus on three aspects of plant-environment interactions. The role of plant hormones in nutrients acquisition is covered in three articles addressing the hormonal control of the uptake and assimilation of nitrogen and sulfate. Six articles focus on abiotic stress responses, and discuss in detail the impact of hormonal pathways on the plant adaptation to drought, salt and other environmental stresses. Stress responses caused by pathogens and hormone mediated defense mechanisms are the topic of three articles.

An improvement of plant performance under less favorable environmental conditions is one of the major challenges of plant biologists nowadays. In this regard, the understanding of principles underlying plant adaptation and defense strategies at the molecular, biochemical, genetic and physiological level provides a valuable intellectual basis for the establishment of novel biotechnologies. It offers possibilities of improving stress tolerance of plants, decrease the yield losses as a result of flooding and infections, and reduce the amount of fertilizers and pesticides applied in the fields.

We greatly appreciate the contribution of all authors and manuscript reviewers, who are all experts in this field. My Guest Editor colleagues José Dinneny, Rodrigo Gutiérrez, Linda Walling and I also truly appreciate the encouragement and help of the Editor-in-Chief and the Editorial Office staff of *Plant Molecular Biology*. Without their support, this specific issue could not have been possible.

